# Evaluating the Velocity and Extent of Cortical Venous Filling in Patients With Severe Middle Cerebral Artery Stenosis or Occlusion

**DOI:** 10.3389/fneur.2021.610658

**Published:** 2021-04-08

**Authors:** Jia'Xing Lin, Zhong'Yuan Cheng, Ying'Ying Shi, Xiang'Ran Cai, Li'An Huang

**Affiliations:** ^1^Department of Neurology, The First Affiliated Hospital, Jinan University, Guangzhou, China; ^2^Medical Imaging Center, The First Affiliated Hospital, Jinan University, Guangzhou, China

**Keywords:** cortical venous filling, middle cerebral artery, severe stenosis, occlusion, dynamic computed tomography angiography

## Abstract

**Objective:** To investigate the velocity and extent of cortical venous filling (CVF) and its association with clinical manifestations in patients with severe stenosis or occlusion of the middle cerebral artery (MCA) using dynamic computed tomography angiography (CTA).

**Methods:** Fifty-eight patients (36 symptomatic and 22 asymptomatic) with severe unilateral stenosis (≥70%) or occlusion of the MCA M1 segment who underwent dynamic CTA were included. Collateral status, antegrade flow, and CVF of each patient were observed using dynamic CTA. Three types of cortical veins were selected to observe the extent of CVF, and the absence of CVF (CVF-) was recorded. Based on the appearance of CVF in the superior sagittal sinus, instances of CVF, including early (CVF_1_), peak (CVF_2_), and late (CVF_3_) venous phases, were recorded. The differences in CVF times between the affected and contralateral hemispheres were represented as rCVFs, and CVF velocity was defined compared to the median time of each rCVF.

**Results:** All CVF times in the affected hemisphere were longer than those in the contralateral hemisphere (p < 0.05). Patients with symptomatic MCA stenosis had more ipsilateral CVF- (*p* = 0.02) and more delayed CVF at rCVF_2_ and rCVF_21_ (rCVF_2_-rCVF_1_) (*p* = 0.03 and 0.001, respectively) compared to those with asymptomatic MCA stenosis. For symptomatic patients, fast CVF at rCVF_21_ was associated with poor collateral status (odds ratio [OR] 6.42, 95% confidence interval [CI] 1.37–30.05, *p* = 0.02), and ipsilateral CVF- in two cortical veins was associated with poor 3-month outcomes (adjusted OR 0.025, 95% CI 0.002–0.33, *p* = 0.005).

**Conclusions:** Complete and fast CVF is essential for patients with symptomatic MCA stenosis or occlusion. The clinical value of additional CVF assessment should be explored in future studies to identify patients with severe MCA stenosis or occlusion at a higher risk of stroke occurrence and poor recovery.

## Introduction

A series of studies have shown that patients with severe intracranial atherosclerotic stenosis (≥70%) or occlusion are at elevated risk of stroke occurrence and recurrence, regardless of whether the best medical therapy is received (1–3). For patients with symptomatic intracranial stenosis, the gradual development of collateral circulation plays a role in protecting perfusion and stabilizing cerebral blood flow (4, 5), including arterial collateral compensation as well as cerebral venous autoregulation (6). The intracranial venous system, a vital component of the vascular neural network, accounts for up to 70% of the total cerebral blood volume (7). However, vascular assessment in intracranial atherosclerosis is mainly based on arterial collateral recruitment, ignoring the significant element of intracranial venous drainage (8).

In recent years, imaging-based venous biomarkers such as cortical veins have been widely reported to play an essential role in acute ischemic events (7, 9–11). The presence of cortical venous filling (CVF) is related to a reduction in infarct volume and decreased severity of hemiparesis (10). Slow or poor CVF of the affected territory probably represents a delayed transmission of cerebral microcirculation, which is more prevalent in strokes in patients with poor collaterals (12–14). Several studies have demonstrated that the asymmetry of CVF can accurately predict clinical prognosis (15–18). In acute stroke patients with severe intracranial arterial stenosis or occlusion, the asymmetrical prominent cortical vein sign is associated with early neurological deterioration (19). However, there have been no reports on the combined assessment of the extent and velocity of CVF in patients with chronic atherosclerosis.

As a non-invasive technique, dynamic computed tomography angiography (CTA)/whole-brain CT perfusion (CTP) is a potential adjunct to traditional digital subtraction angiography (DSA) if time-resolved imaging is required (20). Dynamic CTA/CTP is widely used to evaluate vascular filling from arterial to venous phases because both the velocity and the extent of vessel filling can be considered at the same time, showing high diagnostic accuracy (21–27). To our knowledge, cortical veins, such as the superficial middle cerebral vein (SMCV) and the veins of Trolard (VOT) and Labbe (VOL), receive drainage from most of the arterial supply territories of the middle cerebral artery (MCA) and drain into the superior sagittal sinus (10, 28, 29). This study aimed to investigate the extent and velocity of these key venous fillings and determine whether there is an association between CVF and clinical manifestations in patients with severe unilateral MCA stenosis or occlusion using dynamic CTA/CTP.

## Materials and Methods

### Subjects

The Ethics Committee of the First Affiliated Hospital of Jinan University approved this study. From January 2018 to March 2020, we prospectively screened consecutive patients in the Department of Neurology of the First Affiliated Hospital of Jinan University, with unilateral MCA M1 segment stenosis (≥70%) or occlusion confirmed by DSA or CTA. These patients were divided into symptomatic and asymptomatic groups. Symptomatic patients were those with ischemic stroke or transient ischemic attack within 2 weeks following the onset of symptoms in the distribution of severe stenotic MCA or occlusion. Asymptomatic patients were considered for inclusion if there was no history of cerebrovascular events related to the internal carotid system but still had unilateral MCA M1 segment stenosis (≥70%) or occlusion detected by DSA or CTA. All patients received antiplatelet medication with aggressive risk factors control after admission. Written informed consent was obtained, and dynamic CTA/CTP examinations were performed for each patient.

Patients with any of the following conditions were excluded: (1) internal carotid artery stenosis (≥50%) or contralateral MCA stenosis (≥50%); (2) previous internal carotid artery or MCA stenting, balloon dilatation, or endarterectomy; (3) non-atherosclerotic vasculopathy, such as dissection, moyamoya disease, or vasculitis; (4) evidence of cardiogenic embolism; (5) poor image quality hindering further image analysis; and (6) CT examination-related contraindications. For symptomatic patients, the National Institutes of Health Stroke Scale (NIHSS) score was assessed at the time of admission, and the modified Rankin score (mRS) was obtained at 3 months by telephone interview or outpatient visit.

### CT Protocol

**Figure 1:**
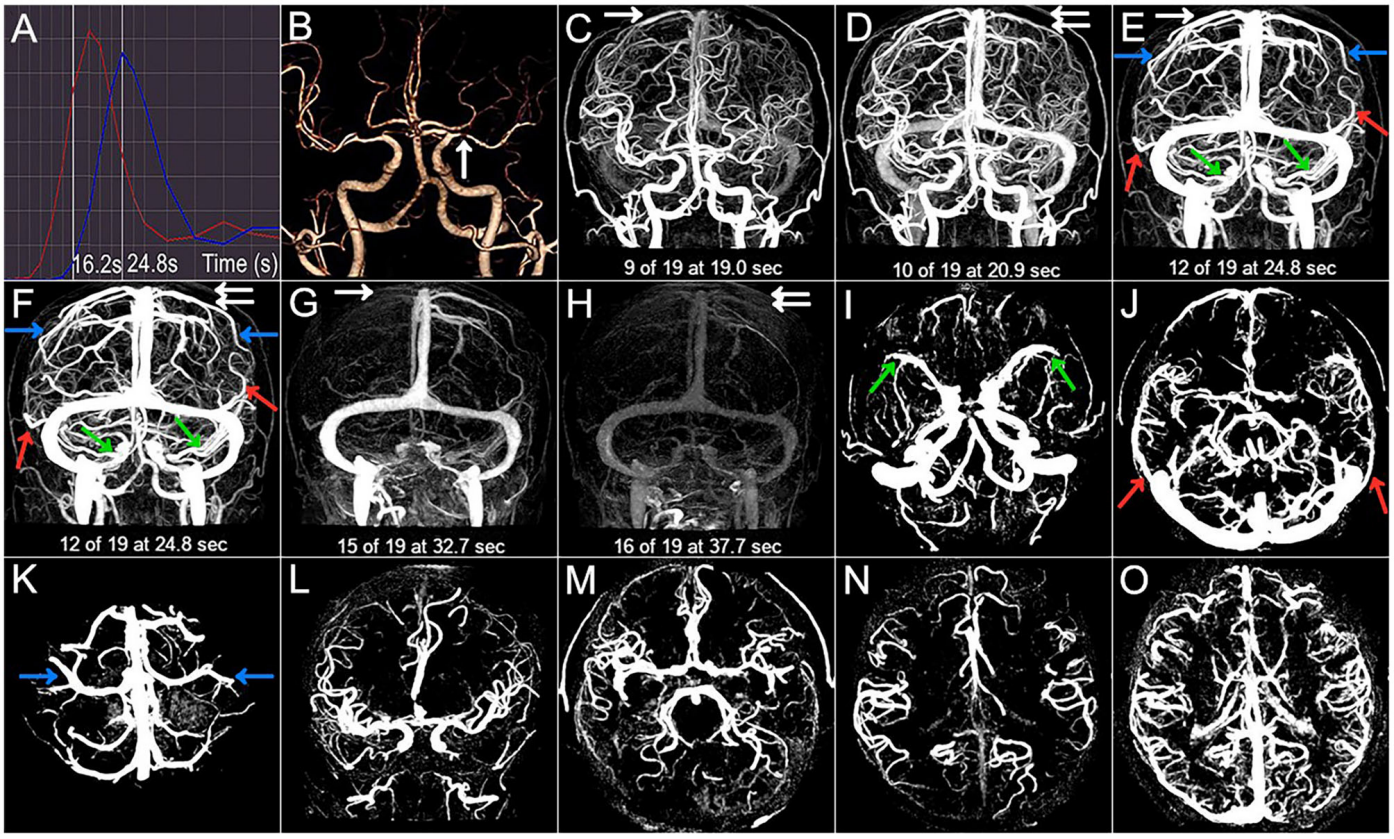
Case 1. A 66-year-old man with a history of hypertension and lipid disorder presented with dizziness for 3 days. **(A)** The arterial and venous time attenuation curves (TACs; red and blue, respectively). The selected arterial phase on TAC (A-TAC) was 16.2 s, and the time-to-peak on the venous TAC (V-TAC) was 24.8 s. **(B)** The arrow points to the left MCA M1 severe stenosis at A-TAC on three-dimensional (3D) computed tomographic angiography (CTA). **(C–H)** The 3D computed tomography venography (CTV) shows cortical venous filling (CVF) draining into the superior sagittal sinus at early (CVF_1_), peak (CVF_2_), and late venous phases (CVF_3_) in the affected (double white arrow) and contralateral (white arrow) hemispheres. Cortical veins begin to be visible in the contralateral (C, CVF_1_, 19.0 s) and affected hemispheres (D, CVF_1_, 20.9 s). The maximum contrast opacification of all cortical veins in the contralateral (E, CVF_2_, 24.8 s) and affected hemispheres (F, CVF_2_, 24.8 s) appear at the same time, and contrast medium in all cortical veins disappears in the contralateral (G, CVF_3_, 32.7 s) and affected hemispheres (H, CVF_3_, 37.7 s). CVF_21_ and CVF_31_ of the contralateral hemisphere are 5.8 s and 13.7 s, respectively, while the CVF_21_ and CVF_31_ of the affected hemisphere are 3.9 s and 16.8 s, respectively. The mean difference between the affected and contralateral hemispheres is 1.9 s for rCVF_1_, 0 s for rCVF_2_, 5 s for rCVF_3_,−1.9 s for rCVF_21_, and 3.1 s for rCVF_31_. The presence (color arrow) and absence (circle) of SMCV (green), VOL (red), and VOT (blue) across all whole venous phases (marked as SMCV+/VOL+/VOT+ and SMCV-/VOL-/VOT-, respectively) are displayed in the 3D CTV and **(I–K)** axial planes of the V-TAC. SMCV-, VOL-, and VOT- are not found in the bilateral hemispheres. **(L,M)** Antegrade flow assessment at TAC in the coronal and axial planes. Contrast filling of the MCA M1 segment and its distal branches in the affected hemisphere is more than two-thirds of the contralateral hemisphere. the contralateral hemisphere, and antegrade flow is preserved. **(N,O)** Collateral status assessment at A-TAC and V-TAC in the axial plane. Complete contrast enhancement of collateral flow at V-TAC in the affected hemisphere with good collateral status.

All patients underwent dynamic CTA/CTP examination with a 320-slice multidetector (Aquilion ONE; Cannon Medical Systems, Tokyo, Japan). A total volume of 50 mL of contrast material with an iodine content of 370 mg/mL (Ultravist 370; Bayer, Leverkusen, Germany) was injected at a flow rate of 6 mL/s. The CT scanning parameters were as follows: tube voltage, 80 kV; matrix, 512 × 512; field of view, 320 mm; rotation time, 0.35 s; and collimator, 0.5 mm × 320. A total of 19 whole-brain volume data were obtained for every patient and loaded into a Vitrea Fx 6.3 workstation (Vital Images, Minnetonka, MN). Based on the separation of the arterial and venous time attenuation curves (TACs) using contrast enhancement of the contralateral MCA and the superior sagittal sinus (25), the maximum intensity projection (MIP) images at different phases were reconstructed. We defined the time point with the best contrast opacification of the bilateral MCA, which was less affected by cortical veins and venous sinuses as the arterial phase (A-TAC) and the time point at the peak points of the venous TAC as the venous phase (V-TAC) (Figure 1A). The stenotic degree of the MCA M1 segment was calculated using 3D CTA with dedicated imaging software (Figure 1B) or verified by DSA (30).

### Image Analysis

Using the reconstructed three-dimensional (3D) CT venography (CTV) MIP images, we observed contrast enhancement of all cortical veins that drained into the superior sagittal sinus. We defined CVF_1_ as the time point when any cortical vein began to appear, CVF_2_ as when most cortical veins reached maximum contrast opacification, and CVF_3_ as the first moment when all cortical veins had completely disappeared (13). In addition, the difference between CVF_2_ and CVF_1_ (CVF_21_) represented the early to peak-venous phase, and the difference between CVF_3_ and CVF_1_ (CVF_31_) represented the whole venous phase. We calculated the above CVF times for both hemispheres according to the timing collection of the 19 volumes (Figures 1C–H). Moreover, the difference in CVF times between the affected and contralateral hemispheres was calculated (rCVFs). To further assess CVF velocity, the fast CVF was defined as a point in time that was less than or equal to the median rCVF, and slow CVF was the opposite (13).

To assess the extent of CVF, we first observed contrast filling of the three cortical veins on 3D CTV, including SMCV, VOT, and VOL. Subsequently, we assessed the MIP reconstruction of the cortical veins above the V-TAC in the axial plane (Figures 1I–K). The presence of CVF at any time point in the venous phase was defined as CVF+ (SMCV+/VOL+/VOT+), whereas the absence of CVF during the whole venous phase was defined as CVF- (SMCV-/VOL-/VOT-) (31). Because CVF- could be seen in the unaffected hemisphere in subjects with anatomical variations (32), we defined the condition of CVF- in the affected hemisphere and CVF+ in the contralateral hemisphere as ipsilateral CVF-. If there was ipsilateral CVF-, the type and number of ipsilateral CVF- were recorded in symptomatic and asymptomatic patients.

The antegrade flow across the stenotic MCA was evaluated in both the coronal and axial planes at A-TAC by referring to the thrombolysis in cerebral infarction scale based on DSA (33, 34) (Figures 1L,M). We reported antegrade flow as preserved or compromised according to whether the vessel filling of the MCA in the affected hemisphere was more than two-thirds of the contralateral hemisphere. Moreover, the collateral status in the affected hemisphere was evaluated at the level of the basal ganglia and thalamus in the axial plane at A-TAC and V-TAC by comparing it with that in the contralateral hemisphere (35) (Figures 1N,O). For our analysis, we reported good collateral status if the collaterals presented complete contrast enhancement at V-TAC or A-TAC and poor collateral status if no contrast enhancement or peripheral contrast enhancement was observed with V-TAC or A-TAC. Two experienced neuroradiologists (Z.Y.C and X.R.C), blinded to all clinical information, independently interpreted and measured the imaging data of all patients. In case of disagreements further judgment was made by consulting a neuroimaging radiologist with higher qualifications.

### Statistical Analyses

Statistical analyses were conducted using SPSS version 21.0 (IBM Corp., Armonk, NY). Variables conforming to the contralateral distribution were reported as mean ± standard deviation, and a *t*-test was conducted for comparison between groups. Categorical variables were expressed as frequencies, and Pearson's chi-square test was used for comparisons between groups. The time from symptom onset or admission to the dynamic CTA/CTP examination, NIHSS score at admission, and CVF times were expressed as the median of the interquartile range (IQR) and were compared using the Mann-Whitney *U* test between groups. To study the relationship between CVF, collateral status, and clinical outcome in the symptomatic group, univariate and multivariate logistic models were used. Results are expressed as odds ratios (ORs) with 95% CIs. *P*-values of <0.05 were considered as statistically significant.

## Results

### Patient Characteristics

**Figure 2:**
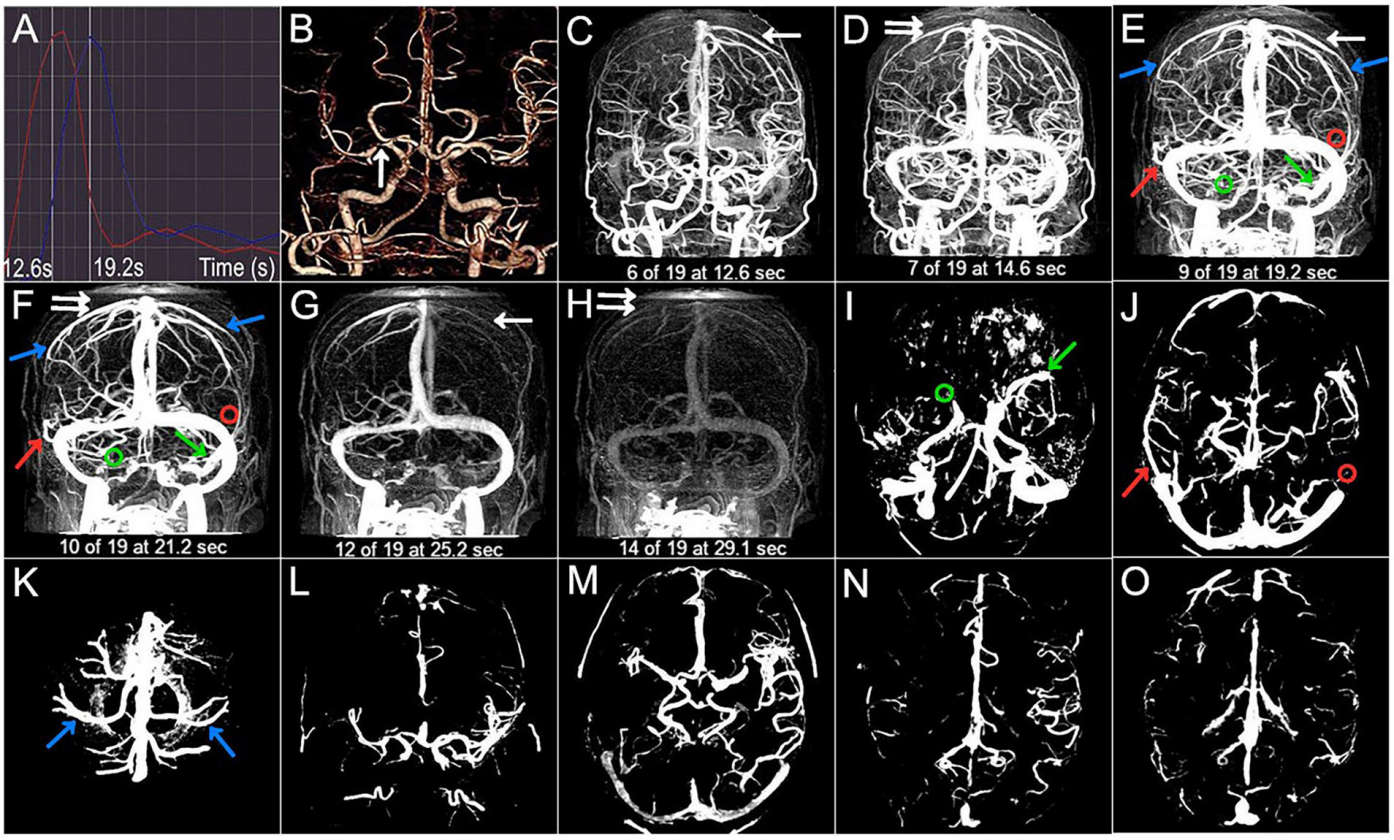
Case 2. A 54-year-old woman with a history of hypertension and diabetes presented with left-sided and left facial droop hemiparesis. The NIHSS score was 5 on admission. 3 months mRS score was 1 (good outcome). **(A)** The selected arterial and venous phases are 12.6 s and 19.2 s, respectively. **(B)** The arrow points to the right M1 severe stenosis. **(C–H)** The CVF_1_, CVF_2_, CVF_3_, CVF_21_, and CVF_31_ of the contralateral hemisphere (white arrow) are 12.6 s, 19.2 s, 25.2 s, 6.6 s, and 12.6 s, respectively, while the CVF_1_, CVF_2_, CVF_3_, CVF_21_, and CVF_31_ of the affected hemisphere (double white arrow) are 14.6 s, 21.2 s, 29.1 s, 6.6 s, and 14.5 s, respectively. The mean difference between the affected and contralateral hemisphere is 0 s for rCVF_21_. **(C–K)** The presence (color arrow) and the absence (circle) of SMCV (green), VOL (red), and VOT (blue). SMCV- is found in the affected hemisphere, and VOL- is found in the contralateral hemisphere. **(L–O)** Compromised antegrade flow and poor collateral status.

**Figure 3:**
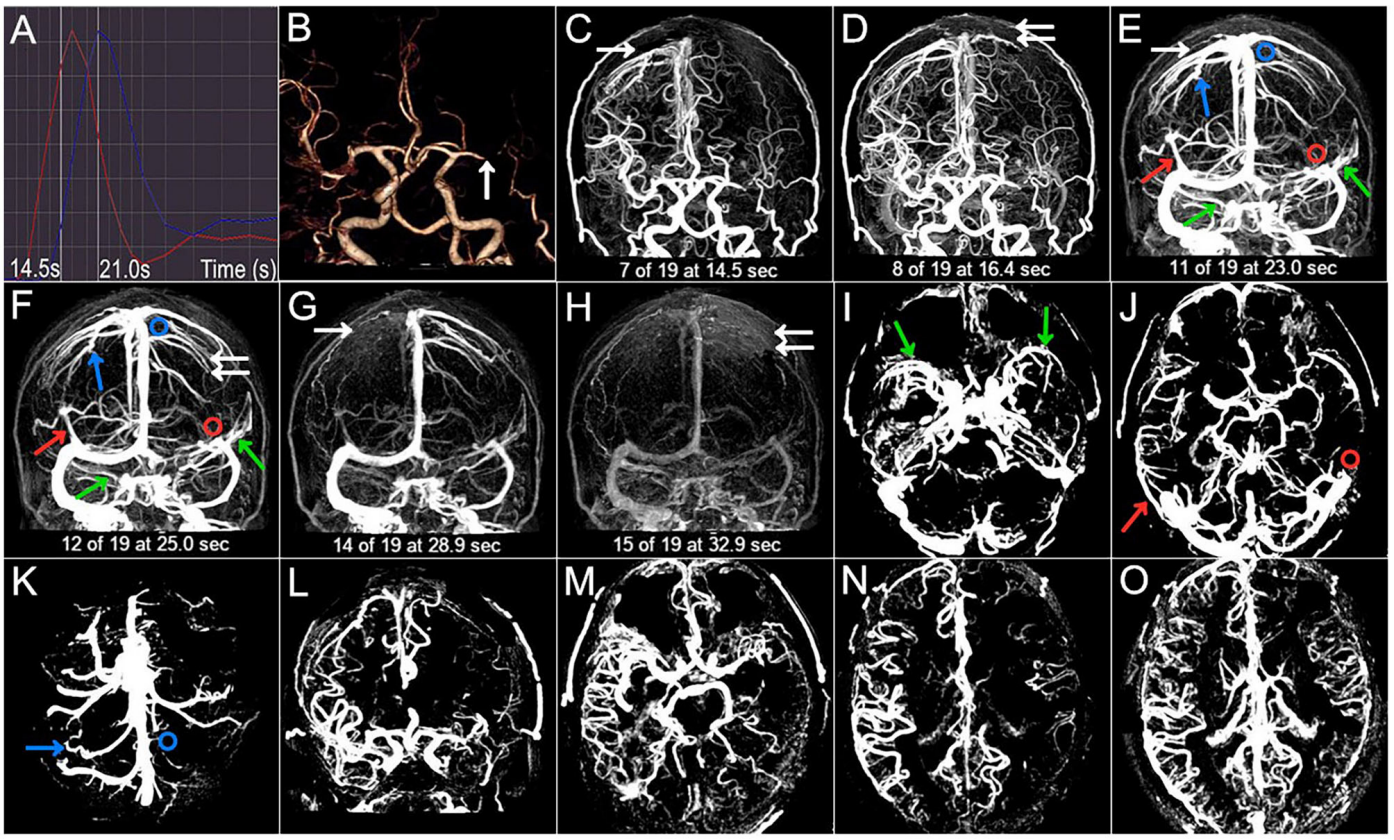
Case 3. A 50-year-old man with a history of hypertension, diabetes, and smoking presented with dysarthria and right-sided hemiparesis. NIHSS score was 6 on admission and 3-month mRS score was 3 (poor outcome). **(A)** The selected arterial phase and the venous phase are 14.5 s and 21.0 s, respectively. **(B)** The arrow points to the left M1 occlusion. **(C–H)** The CVF_1_, CVF_2_, CVF_3_, CVF_21_, and CVF_31_ of the contralateral hemisphere (white arrow) are 14.5 s, 23.0 s, 28.9 s, 8.5 s, and 14.4 s, respectively, while CVF_1_, CVF_2_, CVF_3_, CVF_21_, and CVF_31_ of the affected hemisphere (double white arrow) are 16.4 s, 25.0 s, 32.9 s, 8.6 s, and 16.5 s, respectively. The mean difference between the affected and contralateral hemisphere is 0.1 s for rCVF_21_. **(C–K)** SMCV-, VOL-, and VOT- are not found in the contralateral hemisphere (color arrow), while VOL- (red circle) and VOT- (blue circle) are shown in the affected hemisphere. **(L–O)** Compromised antegrade flow and poor collateral status.

A total of 66 consecutive patients underwent dynamic CTA/CTP scanning. Due to poor image quality, eight patients were excluded. Among the 58 patients included in the study, 36 were symptomatic (31 with ischemic stroke in the MCA territory and five with transient ischemic attack) and 22 were asymptomatic. The median time from symptom onset to dynamic CTA/CTP examination of symptomatic patients was 11 days. The traditional risk factors for intracranial atherosclerosis, the stenotic degree of MCA, and the median time from admission to dynamic CTA/CTP scanning were similar between the symptomatic and asymptomatic groups (Table 1). Figure 1 shows a representative asymptomatic patient, and Figures 2, 3 show two representative symptomatic patients.

**Table 1:** Baseline demographics of symptomatic and asymptomatic patients.

	**Symptomatic patients (*n* = 36)**	**Asymptomatic patients (*n* = 22)**	***p***
Age, years	58.3 ± 9.2	61.3 ± 11.0	0.28
Female	9 (25%)	6 (27%)	0.85
HbA_1c_, %	6.7 ± 2.2	6.2 ± 1.3	0.35
LDL cholesterol, mmol/L	2.7 ± 1.1	2.4 ± 0.9	0.14
HDL cholesterol, mmol/L	1.4 ± 1.4	0.9 ± 0.2	0.06
Cholesterol, mmol/L	4.5 ± 1.6	4.1 ± 1.3	0.35
Triglyceride, mmol/L	2.1 ± 1.0	1.6 ± 1.1	0.14
Hypertension	16 (44%)	12 (55%)	0.46
Diabetes mellitus	12 (33%)	7 (32%)	0.91
Smoking history	23 (64%)	10 (46%)	0.17
Drinking	9 (25%)	8 (36%)	0.36
Lipid disorder	26 (72%)	12 (71%)	0.9
Stenosis of MCA			0.56
Severe stenosis (70–99%)	24 (67%)	13 (59%)	
Occlusion (100%)	12 (33%)	9 (41%)	
Time from admission to dynamic CTA/ CTP, days, median (interquartile range)	3 (1–7)	5 (3–7)	0.12

*HbA_1c_, hemoglobin A_1c_; LDL, low-density lipoprotein; HDL, high-density lipoprotein; MCA, middle cerebral artery; CTA, computed tomography angiography; CTP, computed tomography perfusion*.

### Comparison of CVF Between Symptomatic and Asymptomatic Patients

The CVF times and instances of CVF- of the affected and contralateral hemispheres in both symptomatic and asymptomatic patients were compared, and the results are listed in Table 2. In symptomatic patients, CVF-, SMCV-, VOT-, and VOL- in the affected hemisphere were more common than in the contralateral hemisphere (*p* < 0.001, *p* = 0.02, 0.004, and 0.03, respectively), while there was no significant difference in the proportion and type of CVF- between the affected and contralateral hemispheres in the asymptomatic group. In addition, the CVF times of the affected hemisphere were all significantly longer than those of the contralateral hemisphere in both the symptomatic and asymptomatic groups (*p* < 0.05 for all CVF times).

**Table 2:** Instances of CVF- and CVF times of the affected and contralateral hemispheres.

	**Symptomatic patients (*****n*** **=** **36)**			**Asymptomatic patients (*****n*** **=** **22)**		
	**Affected hemisphere**	**Contralateral hemisphere**	***p***	**Affected hemisphere**	**Contralateral hemisphere**	***p***
Instances of CVF-						
CVF-	19 (53%)	2 (6%)	<0.001	4 (18%)	5 (23%)	1
SMCV-	7 (19%)	0 (0%)	0.02	1 (5%)	1 (5%)	1
VOT-	9 (25%)	0 (0%)	0.004	2 (9%)	0 (0%)	0.47
VOL-	10 (28%)	2 (6%)	0.03	1 (5%)	4 (18%)	0.34
CVF times, s, medians (interquartile range)						
CVF_1_	14.7 (13.2–17.9)	12.8 (11.5–14.5)	<0.001	14.2 (10.0–17.0)	12.2 (9.8–14.5)	<0.001
CVF_2_	21.3 (19.7–26.8)	19.1 (17.3–23.0)	<0.001	21.5 (17.8–23.3)	18.5 (16.9–22.0)	<0.001
CVF_3_	29.0 (24.8–33.1)	25.6 (23.2–30.0)	<0.001	30.9 (26.6–33.5)	26.9 (23.0–30.3)	<0.001
CVF_21_	8.8 (6.6–11.7)	6.6 (5.0–7.9)	<0.001	6.3 (4.8–8.3)	6.0 (4.6–8.3)	0.003
CVF_31_	12.9 (11.8–16.0)	12.5 (7.8–16.3)	0.04	15.3 (13.7–19.4)	14.4 (12.4–16.9)	0.02

*CVF-, absence of cortical venous filling; SMCV-, absence of the superficial middle cerebral vein; VOT-, absence of the vein of Trolard; VOL-, absence of the vein of Labbé; CVF, cortical venous filling; CVF_1_, early venous phase; CVF_2_, peak venous phase; CVF_3_, late venous phase; CVF_21_, early to peak-venous phase; CVF_31_, whole venous phase*.

Imaging findings of symptomatic and asymptomatic patients are listed in Table 3. Since there was no ipsilateral SMCV-, VOT-, or VOL- at the same time in either group, we divided the number of instances of ipsilateral CVF- into two groups: CVF- = 1 and CVF- = 2. Patients with symptomatic MCA stenosis had longer CVF times at rCVF_2_ and rCVF_21_ (*p* = 0.03 and 0.001, respectively; e.g., 0 s in Figure 1 vs. 2 s in Figures 2, 3 for rCVF_2_; −1.9 s in Figure 1 vs. 0 s in Figure 2, 0.1 s in Figure 3 for rCVF_21_) and more ipsilateral CVF- (*p* = 0.02; e.g., ipsilateral CVF+ in Figure 1 vs. ipsilateral CVF- in Figures 2, 3) in the MCA territory of the affected hemisphere, but were similar in the type and number of ipsilateral CVF- compared to the asymptomatic group. In addition, there was no significant difference in collateral status or antegrade flow between the groups.

**Table 3:** Imaging findings in symptomatic and asymptomatic patients.

	**Symptomatic patients (*n* = 36)**	**Asymptomatic patients (*n* = 22)**	***p***
Ipsilateral CVF-	19 (53%)	4 (18%)	0.02
Type of ipsilateral CVF-			
SMCV-	7 (19%)	1 (5%)	0.23
VOT-	9 (25%)	2 (9%)	0.25
VOL-	10 (28%)	1 (5%)	0.07
Number of ipsilateral CVF-			
CVF- = 1	12 (33%)	4 (18%)	0.34
CVF- = 2	7 (19%)	0 (0%)	0.07
CVF times, s, medians (interquartile range)			
rCVF_1_	2.0 (1.2–3.0)	1.8 (0.3–2.0)	0.16
rCVF_2_	2.5 (1.9–3.9)	2.0 (0.8–2.6)	0.03
rCVF_3_	2.7 (0.3–4.0)	2.1 (0.0–4.0)	0.79
rCVF_21_	1.9 (0.4–4.5)	0.1 (0.0–0.5)	0.001
rCVF_31_	1.7 (−1.9–4.7)	0.2 (0.0–2.2)	0.5
Collateral status			0.2
Good	22 (61%)	17 (77%)	
Poor	14 (39%)	5 (23%)	
Antegrade flow			0.78
Preserved	15 (42%)	10 (45%)	
Compromised	21 (58%)	12 (55%)	

*CVF-, absence of cortical venous filling; SMCV-, absence of the superficial middle cerebral vein; VOT-, absence of the vein of Trolard; VOL-, absence of the vein of Labbé; CVF- = 1, absence of one cortical vein; CVF- = 2, absence of two cortical veins; CVF, cortical venous filling; rCVF_1_, relative difference in the early venous phase; rCVF_2_, relative difference in the peak venous phase; rCVF_3_, relative difference in the late venous phase; rCVF_21_, relative difference in the early to peak-venous phase; rCVF_31_, relative difference in the whole venous phase*.

### CVF Velocity and Collateral Status in Symptomatic Patients

It can be concluded from Table 3 that the mean difference between the affected and contralateral hemisphere was 2.0 s for rCVF_1_, 2.5 s for rCVF_2_, 2.7 s for rCVF_3_, 1.9 s for rCVF_21_, and 1.7 s for rCVF_31_ in symptomatic patients. Therefore, we selected fast rCVF_21_ if the difference in CVF time was ≤1.9 s compared to that in the contralateral hemisphere. In patients with symptomatic MCA stenosis, 22 had good collateral status and 14 had poor collateral status. The relationship between CVF velocity and collateral status at each time point is shown in Table 4. Fast CVF at rCVF_21_ was present in 8 (36%) patients with good collateral status, whereas it was found in 11 (79%) patients with poor collateral status (*p* = 0.02). In univariate analysis, fast CVF (only at rCVF_21_, i.e., early to peak-venous phase) was positively associated with poor collateral status (OR 6.42, 95% CI 1.37–30.05, *p* = 0.02; e.g., fast CVF at rCVF_21_ and poor collateral status in Figures 2, 3).

**Table 4:** Relationship between CVF velocity and collateral status in symptomatic patients.

**Good collateral status Poor collateral status**						
	**(*****n*** **=** **22)**	**(*****n*** **=** **14)**	***p***	**OR**	**95% CI**	***p***
CVF velocity						
rCVF_1_			1			
Fast	14 (64%)	8 (64%)				
Slow	8 (36%)	5 (36%)				
rCVF_2_			0.31			
Fast	9 (41%)	8 (64%)				
Slow	13 (59%)	5 (36%)				
rCVF_3_			0.09			
Fast	8 (36%)	10 (71%)				
Slow	14 (64%)	4 (29%)				
rCVF_21_			0.02	6.42	1.37-30.05	0.02
Fast	8 (36%)	11 (79%)				
Slow	14 (64%)	3 (21%)				
rCVF_31_			0.74			
Fast	11 (50%)	8 (57%)				
Slow	11 (50%)	6 (43%)				

*CVF, cortical venous filling; rCVF_1_, relative difference in the early venous phase; rCVF_2_, relative difference in the peak venous phase; rCVF_3_, relative difference in the late venous phase; rCVF_21_, relative difference in the early to peak-venous phase; rCVF_31_, relative difference in the whole venous phase*.

### CVF and Neurological Outcomes at 3 Months in Symptomatic Patients

At 3 months after discharge, 25 patients had a favorable prognosis (mRS score 0–2), while 11 had a poor outcome (mRS score >2). Table 5 shows the associations between clinical and imaging variables and clinical outcomes at the 3-month follow-up. There was no significant relationship between baseline characteristics and clinical prognosis. The proportion of patients with poor outcomes was greater in those with higher NIHSS scores after admission (*p* = 0.04). Four patients underwent elective endovascular angioplasty for severe stenotic MCA within 3 months; however, there was a non-significant trend toward a good prognosis. Ipsilateral CVF-, type of ipsilateral CVF-, absence of filling of one cortical vein, poor collateral status, and compromised antegrade flow were not significantly related to poor clinical outcomes. Among the 11 patients with poor outcomes, the absence of filling of the two cortical veins was found in six cases (56%) (*p* < 0.001). In univariate analysis, the absence of filling of the two cortical veins was associated with clinical results (OR 0.04, 95% CI 0.003–0.36, *p* = 0.005). Furthermore, multivariate analysis showed that the absence of filling of the two cortical veins was still related to the poor outcome at the 3-month follow-up (adjusted OR 0.025, 95% CI, 0.002–0.33, *p* = 0.005) (Figure 2 vs. Figure 3).

**Table 5:** Univariate associations of baseline characteristics and clinical outcomes at 3 months.

	**Good outcomes (*n* = 25)**	**Poor outcomes (*n* = 11)**	***p***	**OR**	**95% CI**	***p***
Age, years	57.0 ± 9.2	61.5 ± 8.6	0.18			
Hypertension	10 (40%)	6 (55%)	0.48			
Diabetes	7 (28%)	5 (46%)	0.45			
Smoking history	16 (64%)	7 (64%)	1			
Drinking	6 (24%)	3 (27%)	1			
Lipid disorder	18 (72%)	8 (73%)	1			
NIHSS, median (interquartile range)	3 (2–4)	5 (2–8)	0.04	1.36	1.00–1.86	0.05
Angioplasty	3 (12%)	1 (9%)	1			
Ipsilateral CVF-	11 (44%)	8 (73%)	0.16			
Ipsilateral SMCV-	4 (16%)	3 (27%)	0.65			
Ipsilateral VOT-	6 (24%)	3 (27%)	0.57			
Ipsilateral VOL-	5 (20%)	5 (45%)	0.12			
CVF- = 1	9 (36%)	3 (27%)	0.71			
CVF- = 2	1 (4%)	6 (56%)	<0.001	0.04	0.003–0.36	0.005
Slow rCVF_1_	11 (44%)	2 (18%)	0.26			
Slow rCVF_2_	12 (48%)	6 (55%)	1			
Slow rCVF_3_	11 (44%)	7 (64%)	0.47			
Slow rCVF_21_	11 (44%)	6 (55%)	0.41			
Slow rCVF_31_	11 (44%)	6 (55%)	0.72			
Poor collateral status	11 (44%)	3 (27%)	0.47			
Compromised antegrade flow	13 (52%)	8 (73%)	0.3			

*NIHSS, National Institutes of Health Stroke Scale; CVF-, absence of cortical venous filling; SMCV-, absence of the superficial middle cerebral vein; VOT-, absence of the vein of Trolard; VOL-, absence of the vein of Labbé; CVF- = 1, absence of one cortical vein; CVF- = 2, absence of two cortical veins; rCVF_1_, relative difference in the early venous phase; rCVF_2_, relative difference in the peak venous phase; rCVF_3_, relative difference in the late venous phase; rCVF_21_, relative difference in the early to peak-venous phase; rCVF_31_, relative difference in the whole venous phase*.

## Discussion

To the best of our knowledge, this is the first prospective study to describe the velocity and extent of CVF in patients with severe stenosis or occlusion of the MCA responsible or not responsible for recent ischemic stroke or transient ischemic attack. Prolonged CVF times were commonly found at different stages of the venous phase in the affected hemisphere. Patients with symptomatic MCA stenosis also had longer CVF times and more ipsilateral CVF- than those with asymptomatic MCA stenosis. Moreover, our preliminary study demonstrated that fast CVF was associated with poor collateral status, and the absence of filling of the two cortical veins was linked with poor outcome, suggesting the essential and irreplaceable role of cortical veins in patients with symptomatic high-grade MCA stenosis or occlusion.

In this study, we noticed an obvious relationship between delayed filling of the ipsilateral cortical veins and severe stenosis or occlusion of the MCA. Adequate collateral perfusion requires arterial and venous autoregulation to redistribute cerebral blood flow and maintain cerebral perfusion (6, 7), which might indicate a slowdown of venous drainage to varying degrees in response to chronic cerebral hypoperfusion. However, there was a similar proportion of CVF- in the bilateral hemispheres of asymptomatic patients. A possible explanation is that compensatory venous collaterals can extensively communicate at the cortical surface (36, 37), resulting in delayed venous drainage, but the presence of cortical vein collaterals (SMCV, VOT, VOL).

Similar to previous research studies on acute MCA occlusion (10, 38), our study demonstrated that symptomatic patients were more likely to experience slower and asymmetrical CVF in the affected MCA territory. No serial studies have assessed changes in the cortical veins over time after qualifying ischemic events. If ischemic strokes occur under the condition of chronic stenosis, a compensatory hemodynamic function of venous collaterals associated with increased venous blood volume and cerebral vasodilation may be continuously and seriously impaired (39), resulting in slower or even absent ipsilateral CVF for a long time. Additionally, a previous study has shown that VOL and VOT are often seen in a certain hemisphere in contralateral subjects (40), which may partially explain why the type and number of ipsilateral CVF- between the symptomatic and asymptomatic groups were not enough to contribute to a statistical difference.

We also found that fast CVF was closely related to poor arterial collateral in symptomatic patients. This finding was not in line with that of a previous study, which demonstrated a trend toward slow CVF with a worse collateral grade in patients with acute MCA occlusion (10). It is important to mention that the clinical correlations of arterial collaterals in this study were not evident, indicating that the rapid and effective drainage of cortical veins may be beneficial in compensating for potential arterial hemodynamic damage. Moreover, once chronic atherosclerosis reaches a stage with severe stenosis or complete occlusion, it will lead to insufficient and slow venous drainage far beyond the nearby arterial collaterals, even if good collateral flow tends to compensate circulation. Interestingly, delayed drainage of cortical veins in the early to peak-phase, not the late venous phase (41), was related to arterial collateral status and stroke occurrence in this study. The CVF time lag in the affected hemisphere has been proven to be associated with prolonged mean transit time (12), which probably reflects compromised perfusion through microcirculation at an earlier stage of venous drainage due to progressive microvascular obstruction (16).

Our results support the effect of asymmetric CVF on the prognosis of ischemic stroke demonstrated in previous studies (15–18). In contrast to acute occlusion, the number of ipsilateral CVF-, rather than the type of ipsilateral CVF- has superior prognostic value in patients with symptomatic MCA stenosis in this study, which might be explained by hemodynamic mechanisms. First, the lower extent of CVF during chronic stenosis may be explained by the upregulation of vascular endothelial cell adhesion molecules and the downregulation of tight junction proteins to weaken the blood-brain barrier in hypoperfusion (42). Other explanations include active venous contraction (43), leukocyte-platelet aggregation obstruction (44, 45), and passive thin-walled venule compression (46). In addition, the respective collateral pathways of venous drainage are irrevocably impaired when the number of ipsilateral CVF- is high (47). Furthermore, the severely impaired venous drainage pathway around the lesions, accompanied by long-term cerebral hypoperfusion, will ultimately damage the required perfusion and upstream arterial regulation (48), causing subsequent pathophysiological consequences that are difficult to correct.

In conclusion, we used dynamic CTA/CTP to investigate the relationships between various stages of cortical venous flow, symptom occurrence, and clinical prognosis in the present study. An increased proportion of CVF- or prolonged CVF times in the early to peak-phase in the affected hemisphere are more likely to be associated with recent ischemic events in patients with severe MCA stenosis or occlusion. Moreover, a lower extent of CVF is associated with worse short-term clinical outcomes, and fast CVF is likely to be a reaction to poor collateral flow, suggesting the importance of complete and fast cortical venous drainage in symptomatic MCA stenosis. Further prospective studies are warranted to validate the feasibility of CVF assessment in identifying patients with high-grade MCA stenosis or occlusion at a higher risk of stroke occurrence and poor prognosis.

However, this study had several shortcomings. First, the sample size collected in this study was small. Larger sample sizes will be critical for moving the field forward. Second, the period between symptom onset and imaging acquisition could not be determined for asymptomatic patients without clinical symptoms. Third, CVF-related MIP images were acquired in the target subjects with unilateral MCA severe stenosis or occlusion, which may be difficult to rule out patients with multifocal intracranial atherosclerotic stenosis. Therefore, a contralateral MCA with <50% stenosis was used as a control. Fourth, there is a certain proportion of CVF- in healthy individuals. Considering the physiological differences in each patient, our study mainly focused on the asymmetry of CVF in the affected hemisphere and evaluated whether ipsilateral CVF- had any effect on the occurrence and prognosis of stroke. Finally, the time interval of commonly used clinical image acquisition was quite long because of the clinical limitations of dynamic CTA/CTP. Therefore, it is necessary to carefully compare the CVF times at different stages of venous drainage.

## Data Availability Statement

The raw data supporting the conclusions of this article will be made available by the authors, without undue reservation.

## Ethics Statement

The studies involving human participants were reviewed and approved by The Ethics Committee of the First Affiliated Hospital of Jinan University. The patients/participants provided their written informed consent to participate in this study.

## Author Contributions

LH contributed to the conception and design of the study and edited the manuscript. JL performed data analyses and wrote the manuscript. JL and YS contributed toward the patient recruitment. ZC and XC interpreted and measured the imaging data. All authors contributed to the article.

## Conflict of Interest

The authors declare that the research was conducted in the absence of any commercial or financial relationships that could be construed as a potential conflict of interest.
